# Alteration of lipid profile and value of lipids in the prediction of the length of hospital stay in COVID‐19 pneumonia patients

**DOI:** 10.1002/fsn3.1907

**Published:** 2020-10-27

**Authors:** Chen Qin, Huang Minghan, Zhao Ziwen, Luo Yukun

**Affiliations:** ^1^ Department of Cardiology Fujian Medical University Union Hospital Fuzhou China; ^2^ Fujian Institute of Coronary Artery Disease Fuzhou China; ^3^ Fujian Heart Medical Center Fuzhou China; ^4^ Department of Gastroenterology The Second People’s Hospital Affiliated to Fujian University of Traditional Chinese Medicine Fuzhou China

**Keywords:** cholesterol, COVID‐19 pneumonia, infection, length of hospital stay, lipid profile

## Abstract

To observe lipid profiles and their alterations in hospitalized patients with COVID‐19 pneumonia (NCP) and evaluate the value of lipids for the prediction of the length of hospital stay (LOS), a total of 248 patients aged 18 years or older were enrolled in this retrospective study. At admission, the median levels of triglyceride (TG), total cholesterol (TC), high‐density lipoprotein cholesterol (HDL‐C), and low‐density lipoprotein cholesterol (LDL‐C) in all patients were 1.11, 4.00, 0.89, and 2.11 mmol/L, respectively. Compared with common cases (*n* = 174), severe cases (*n* = 74) exhibited higher TG and HDL‐C, and lower LDL‐C. Levels of TC and LDL‐C were negatively correlated with LOS. In 68 severe cases, serum lipids were followed up during hospitalization, and the median LOS was 29 days. The average levels of serum lipids were lowest at admission and gradually increased during hospitalization. Compared with the LOS ≤ 29 days group, serum levels of TC, HDL‐C, and LDL‐C were significantly lower in the LOS > 29 days group at admission; this lower trend was found in the subsequent tests for TC and LDL‐C but not for HDL‐C or TG. Multiple‐variant COX regression showed that levels of TC or LDL‐C at admission were independent risk of LOS prolongation. Together, these findings suggest that in patients with NCP, levels of TC and LDL‐C at admission were negatively correlated with LOS. In severe cases, the gradual increase in TC, LDL‐C, and HDL‐C during hospitalization might indicate gradual recovery. TC < 3.75 mmol/L or LDL‐C < 1.7 mmol/L at admission may act as an independent predictor of prolonged LOS.

## Graphical abstract

Lipid levels at admission were low in coronavirus disease 2019 (COVID‐19) infected pneumonia (NCP‐19) patients. Lipid levels gradually increased during the recovery from NCP‐19. Lipid levels of TC, LDL and HDL are inversely connecting with the length of hospital stay in NCP‐19 patients. Monitoring of lipid alteration is important for therapeutic strategy in NCP‐19 patients.

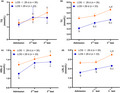

## 1 INTRODUCTION

The outbreak of the novel coronavirus SARS‐CoV‐2 (causing coronavirus disease‐2019; COVID‐19) has spread to most countries in the world. COVID‐19 infection results in diverse clinical outcomes, ranging from no symptoms to critically severe pneumonia with extrapulmonary manifestations (Guan et al., 2020; Huang et al., 2020).

Lipids play numerous indispensable functions in lung biology and the pathophysiology of viral infection. Pulmonary surfactant is over 90% lipids and 10% protein by weight (Han & Mallampalli, 2015). Cholesterol is the major neutral lipid of pulmonary surfactant, and it is involved in suppressing viral infection in the respiratory tract (Fessler & Summer, 2016). Lipid tracer studies have indicated that more than 80% of lung cholesterol is derived from the plasma (Turley et al., 1981). Lipids are also involved in various stages of the virus life cycle; they are essential components of membranes and are thus indispensable for the formation and function of the viral replication complex (Hsu et al., 2010). Lipids are also an integral part of the innate and adaptive immune systems in the context of infections (Wendel et al., 2007).

All lipid parameters are significantly deranged during acute infection. The common lipid alterations include a decrease in total cholesterol (TC) levels and an increase in the concentration of triglyceride‐rich lipoproteins. Additionally, low‐ and high‐density lipoprotein cholesterol (LDL‐C and HDL‐C, respectively), apolipoprotein‐A1, and apolipoprotein‐B levels decrease. These lipid alterations may have a prognostic and diagnostic role in certain infections (Filippas‐Ntekouan et al., 2017). Little is known about the effect of acute viral infection on lipid metabolism, and detailed information on the changes in lipid profiles during COVID‐19 infection is lacking.

The length of hospital stay (LOS) reflects the time of recovery from NCP according to the discharge criteria, and prolongation of hospital stay is associated with the severity and complexity of NCP to some extent. Additionally, a longer LOS is also associated with higher hospitalization costs and more medical resource consumption.

In this study, we aimed to observe the characteristics of lipid alterations during hospitalization in patients with COVID‐19 pneumonia (NCP); a further goal was to evaluate whether there is any predictive value of lipid profiles at admission for LOS.

## 2 METHODS

### 2.1 Study population

Subjects of this retrospective study were patients with NCP aged 18 years or older who were treated at departments of the Tumor Center and Xi Yuan of Union Hospital, Tongji Medical College, Huazhong University of Science and Technology, and departments of Yichang Third People's Hospital, between February 14 and April 5. The diagnosis of NCP and clinical criteria for severe NCP were established by the guidelines of the National Health Commission of China (COVID‐19 pneumonia management guideline (Fifth version)).

In the guideline, clinical cases were described as the following four types: (a) Mild type: mild clinical symptoms, no pneumonia on imaging. (b) Common type: fever, respiratory symptoms, and other symptoms, with imaging manifestations of pneumonia. (c) Severe type: in accordance with any of the following: respiratory distress, RR > 30 beats/min; resting state, oxygen saturation < 93%; PaO_2_/FiO_2_ < 300 mmHg (l mmHg = 0.133 kpa). (d) Critical type: those who meet one of the following conditions: respiratory failure and need mechanical ventilation; shock; combined with other organ failure and need intensive care and treatment. The discharge standards were as follows: body temperature returned to normal for more than 3 days; respiratory symptoms improved significantly; inflammation of the lungs showed obvious signs of absorption; respiratory nucleic acid was negative two consecutive times (minimum 48‐hr interval). All patients were treated according to the national guidelines with antiviral therapy, including intravenous ribavirin 0.5 g twice daily and/or oral arbidol 0.2 g three times daily or lopinavir and ritonavir tablets 2 pills three times daily. Most of the patients took traditional Chinese medicine. Antibiotics were used in patients who had bacterial infections. Some patients also received glucocorticoid and/or intravenous immunoglobulin (IVIG)/albumin administration. In patients who need nutritional support, enteral nutrition, and intravenous nutrition support were given according to the nutritional status of the patients.

### 2.2 Data collection

The following parameters were retrospectively assessed using medical records: age, sex, LOS, and clinical comorbidities such as hypertension, diabetes, coronary heart disease, and statin treatment. Peripheral venous blood samples were obtained after a 12 hr fasting period. Lipid tests included TC, LDL‐C, HDL‐C, and triglycerides (TGs). Blood glucose, hemoglobin, lymphocytes, albumin, alanine aminotransferase (ALT), aspartate aminotransferase (AST), serum creatinine, urea, cardiac troponin I, brain natriuretic peptide (BNP), and C‐reactive protein (CRP) were recorded. Serum biochemical measurements were performed in the hospital clinical laboratory using routine automated techniques.

### 2.3 Statistical analysis

Continuous variables are expressed as the mean ± standard deviation (*SD*) or median (interquartile range), according to their distribution, which was assessed by the Kolmogorov–Smirnov test. Categorical variables are expressed as numbers and relative frequencies. Normally distributed continuous variables were compared with Student's *t* test and one‐way analysis of variance (ANOVA). Nonparametric tests, including chi‐squared, the Kruskal–Wallis H test, and crosstabs, were used to analyze both the non‐normally distributed continuous variables and categorical variables. Correlation analysis was performed using Spearman rank correlation analysis. The optimal cutoff values of serum lipids and age for predicting prolonged LOS in severe cases were calculated based on maximizing the sum of sensitivity and specificity for each index using receiver operating characteristic (ROC) curve analysis. To identify the independent predictors of prolonged LOS among patient‐ or serum‐lipid‐related covariates, hospital discharge was defined as the event, and a Cox proportional hazards model was constructed to calculate the hazard ratio (HR) and 95% confidence interval (CI) to compare the risk of events between groups, classified according to optimal cutoff values or clinical experiences. The univariate screened by COX regression (*p* < .1) was entered into multivariate COX analysis. Variables were carefully chosen to ensure the parsimony of the final models. As the collinearity between different types of serum lipids might interfere with regression results, we established different regression modes with each selected serum lipid variable and other clinical variables, respectively. A two‐sided *p*‐value < .05 was considered statistically significant. All statistical analyses were performed with SPSS 26.0 (IBM, Armonk, NY, USA).

## 3 RESULTS

### 3.1 Baseline characteristics at admission

A total of 254 patients were admitted to our wards, and six patients died during hospitalization: two patients died of a massive cerebral hemorrhage, one patient died of unidentified hypertonic dehydration within 24 hr of admission, and three patients died of multisystem organ failure after being transferred to the intensive care unit. A total of 248 patients were enrolled in the final study cohort. In Hubei at that time, the mild type patients were all treated in mobile cabin hospitals. The patients in our wards were mainly the following three types: the common type (*n* = 174), the severe type (*n* = 68), and the critical type (*n* = 6). In this paper, we classified the severe type and critical type patients into severe cases (*n* = 74).

The baseline characteristics and clinical laboratory data of 248 patients at admission are shown in Table 1. At admission, the median levels of TG, TC, HDL‐C, and LDL‐C were 1.52, 4.00, 0.89, and 2.11 mmol/L, respectively. Compared with common cases (*n* = 174), higher TG and HDL‐C and lower LDL‐C were observed in severe cases (*n* = 74). Serum levels of TC were not significantly different between the two groups. Longer LOS, older age, lower albumin, and higher FBG, AST, urea, and creatinine were observed in the severe group. Other laboratory indicators, such as APOA1, APOB, LP(a), lymphocyte count, hemoglobin concentration, CRP, and BNP, did not reach significant differences between the two groups. A higher proportion of hypertension, coronary heart disease, and diabetes mellitus was observed in the severe group. Nevertheless, the proportion of statin treatment use was not significantly different between the two groups.

**Table 1:** Baseline characteristics at admission

	Total (*n* = 248)	Common cases (*n* = 174)	Severe cases (*n* = 74)	*p*‐Value
Hospital stay (days)^a^	23.0 (11)	22.0 (9)	29.5 (13)	<.001
Age (years)	55.04 ± 15.87	50.93 ± 15.59	64.73 ± 11.88	<.001
Male, *n* (%)^b^	130 (52.4)	91 (52.3)	39 (52.7)	1.000
TG (mmol/L)^a^	1.11 (0.56)	1.10 (0.54)	1.22 (0.59)	.045
TC (mmol/L)^a^	4.00 (1.07)	4.04 (1.14)	3.90 (1.12)	.184
HDL‐C (mmol/L)^a^	0.89 (0.37)	0.86 (0.31)	1.03 (0.49)	<.001
LDL‐C (mmol/L)^a^	2.11 (0.85)	2.20 (0.91)	1.99 (0.75)	.024
APOA1^a^	0.80 (0.23)^c^	0.81 (0.24)^d^	0.73 (0.18)^e^	.120
APOB^a^	0.75 (0.25)^c^	0.76 (0.25)^d^	0.72 (0.18)^e^	.222
LP(a)^a^	108.5 (167.0)^c^	109.0 (182.0)^d^	77.0 (145.0)^e^	.912
Lymphocyte (×10^9^/L)^a^	1.24 (0.69)	1.24 (0.69)	1.28 (0.68)	.895
Hemoglobin (g/L)^a^	121.0 (23.0)	122.0 (23.0)	121.0 (26.0)	.699
ALT (U/L)^a^	23.00 (21.00)	22.00 (23.25)	24 (18.5)	.137
AST (U/L)^a^	23.0 (14.0)	21.0 (12.0)	31.0 (21.0)	<.001
Albumin (g/L)^a^	39.30 (5.70)	39.75 (5.70)	37.90 (7.50)	.003
FBG (mmol/L)^a^	5.21 (1.26)	5.01 (1.00)	5.66 (1.51)	<.001
Urea (mmol/L)^a^	4.20 (2.00)	4.17 (1.83)	4.50 (2.33)	.008
Creatinine (μmol/L)^a^	69.50 (24.00)	65.90 (24.87)	74.00 (22.80)	<.001
CRP (mg/L)^a^	12.70 (29.71)	11.80 (27.80)	14.30 (40.66)	.056
Hypertension, *n* (%)^b^	63 (25.4)	36 (20.7)	27 (36.5)	.011
Diabetes, *n* (%)^b^	31 (12.5)	16 (9.2)	15 (20.3)	.021
CHD, *n* (%)^b^	16 (6.5)	7 (4.0)	9 (12.2)	.024
Previous Statin treatment, *n* (%)^b^	20 (8.1)	10 (5.7)	10 (13.5)	.071

Note

TG, triglyceride; TC, total cholesterol; HDL‐C, high‐density lipoprotein cholesterol; LDL‐C, low‐density lipoprotein cholesterol; APO, apolipoprotein; ALT, alanine aminotransferase; AST, aspartate aminotransferase; BNP, brain natriuretic peptide; CRP, C‐reactive protein; FBG, fasting blood glucose; CHD, coronary heart disease; LP, lipoprotein.

Normally distributed continuous variables are presented as the mean ± standard deviation (*SD*).

^a^ Non‐normally distributed variables are presented, including median and interquartile range (IQR).

^b^ Categorical parameters are represented as total *n* and percentage.

^c^ *n* = 178.

^d^ *n* = 159.

^e^ *n* = 19.

### 3.2 Correlations between LOS and serum lipid levels at admission

**Figure 1:**
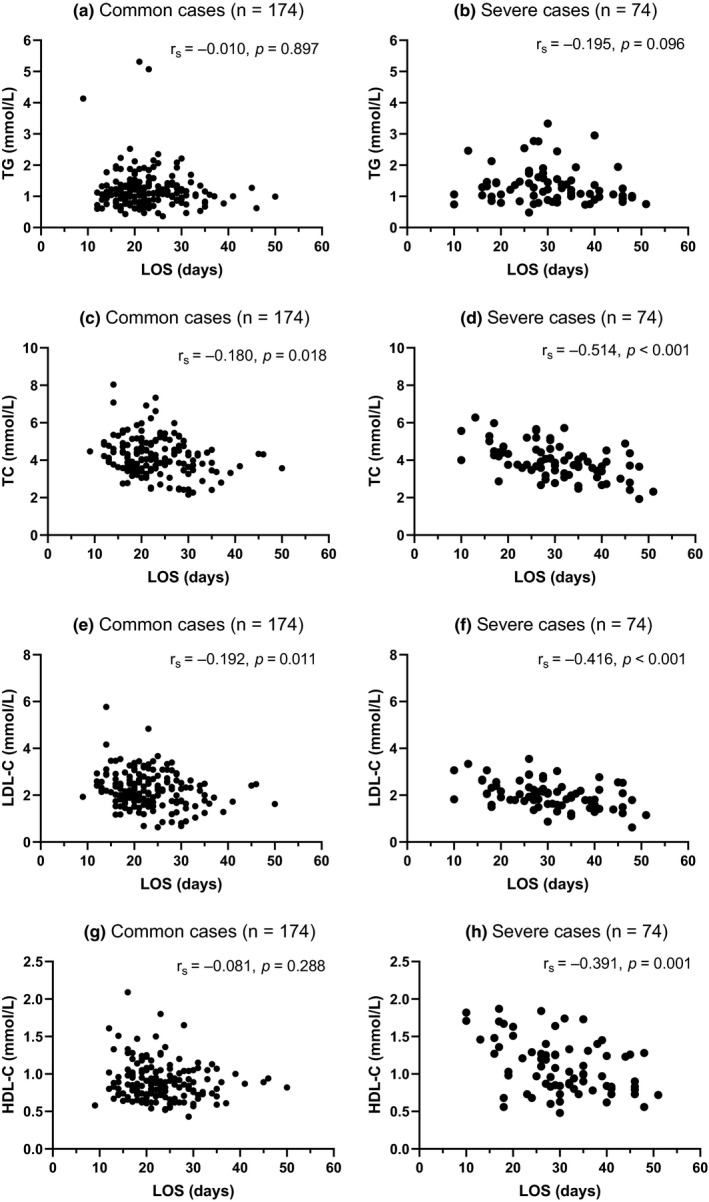
Correlations between LOS and serum lipid levels at admission; (a, b) No significant correlations were observed between serum levels of TG at admission and LOS in common cases or severe cases; (c–f) Serum levels of TC and LDL‐C at admission were negatively correlated with LOS in both common cases and severe cases; (g, h) Serum levels of HDL‐C at admission were negatively correlated with LOS in severe cases but not in common cases

Spearman rank correlation analysis was used to clarify the correlations between LOS and serum lipid levels at admission. As shown in Figure 1, no significant correlations were observed between serum levels of TGs at admission and LOS in both common cases and severe cases (Figure 1a,b). Serum levels of TC and LDL‐C at admission were negatively correlated with LOS in both common cases and severe cases (Figure 1c–f), and serum levels of HDL‐C at admission were negatively correlated with LOS in severe cases but not in common cases (Figure 1g,h).

### 3.3 Changes in serum lipid profiles in severe cases during hospitalization

**Figure 2:**
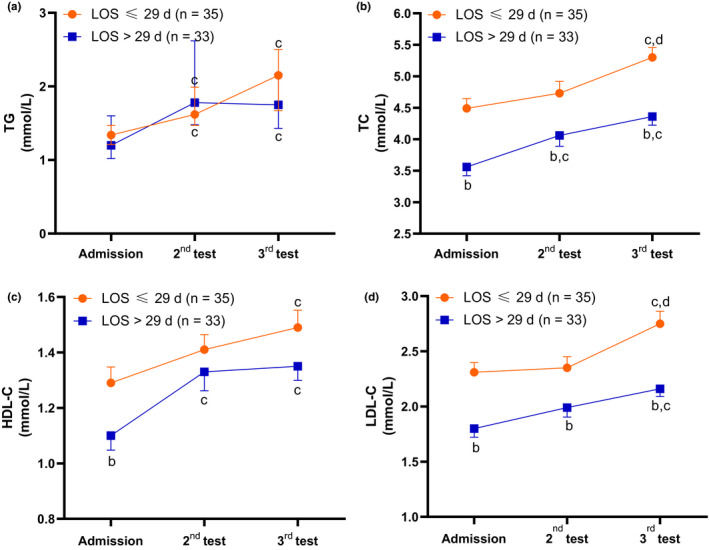
Changes in the serum lipid profile during hospitalization; (a) Data are shown as the median (bar = IQR); (b–d) Data are shown as the mean (bar = standard error); (b) *p* < .05, versus LOS ≤ 29 days group; (c) *p* < .05, versus at admission; (d) *p* < .05, versus 2nd test

To observe the continuous alterations in serum lipids, 68 patients with severe cases who underwent lipid tests three times were included; the interval between each test was 5–10 days. The median LOS was 29 days among those 68 patients, so the patients were further divided into two groups: LOS ≤ 29 days group and LOS > 29 days group. As shown in Table 2 and Figure 2, the average levels of TC, LDL‐C, HDL‐C, and TG at admission in 68 severe cases were as low as 4.04 ± 0.97, 2.06 ± 0.55, 1.20 ± 0.33 mmol/L, and 1.52 (0.72) mmol/L, respectively. Compared with the LOS ≤ 29 days group, the levels of TC, LDL‐C and HDL‐C were significantly lower in the LOS > 29 days group at admission (*p* < .05), and this trend was also shown during the following 2nd and 3rd tests for TC and LDL‐C (both *p* < .05) but not for HDL‐C or TG.

**Table 2:** Changes of serum lipid profiles in severe cases during hospitalization

		Total (*n* = 68)	LOS ≤ 29 days (*n* = 35)	LOS > 29 days (*n* = 33)
TG (mmol/L)^a^	At admission	1.52 (0.72)	1.34 (0.76)	1.20 (0.69)
	2nd test	1.64 (1.25)^c^	1.62 (1.02)^c^	1.78 (1.47)^c^
	3rd test	1.79 (1.20)^c^	2.15 (1.33)^c^	1.75 (0.99)^c^
TC (mmol/L)	At admission	4.04 ± 0.97	4.49 ± 0.91	3.56 ± 0.80^b^
	2nd test	4.40 ± 1.10^c^	4.73 ± 1.11	4.06 ± 0.98^b^, ^c^
	3rd test	4.84 ± 1.03^c^	5.30 ± 1.03^c^, ^d^	4.36 ± 0.78^b^, ^c^
HDL‐C (mmol/L)	At admission	1.20 ± 0.33	1.29 ± 0.34	1.10 ± 0.30^b^
	2nd test	1.37 ± 0.35^c^	1.41 ± 0.32	1.33 ± 0.39^c^
	3rd test	1.42 ± 0.34^c^	1.49 ± 0.37^c^	1.35 ± 0.29^c^
LDL‐C (mmol/L)	At admission	2.06 ± 0.55	2.31 ± 0.52	1.80 ± 0.45^b^
	2nd test	2.18 ± 0.58	2.35 ± 0.60	1.99 ± 0.49^b^
	3rd test	2.46 ± 0.63^c^	2.75 ± 0.67^c^, ^d^	2.16 ± 0.40^b^, ^c^
Treatment				
Glucocorticoids, *n* (%)		19 (25.7)	5 (14.3)	14 (42.4)^b^
IVIG/albumin, *n* (%)		16 (21.6)	5 (14.3)	11 (33.3)
Mechanical ventilation, *n* (%)		6 (8.1)	0 (0.0)	6 (18.2)
Parenteral nutrition support, *n* (%)		11 (16.2)	3 (8.6)	8 (24.2)

Note

Abbreviations as Table 1. IVIG, intravenous immunoglobulin.

Normally distributed continuous variables are presented as the mean ± standard deviation (*SD*).

^a^ Non‐normally distributed variables are presented including median and interquartile range (IQR).

^b^ *p* < .05, versus LOS** ≤ **29 days group.

^c^ *p* < .05, versus at admission.

^d^ *p* < .05, versus 2nd test.

The average levels of TG, TC, HDL‐C, and LDL‐C in 68 severe cases gradually and significantly increased during the following 2nd (except for LDL‐C) and 3rd tests (both *p* < .05). This growing trend also showed significance for both groups in the 3rd test. Compared with the 2nd test, the concentration of the 3rd test for TC and LDL‐C reached even significantly higher levels of 5.30 ± 1.03 and 2.75 ± 0.67 mg/L, respectively, in the LOS ≤ 29 days group.

### 3.4 Discriminative values of serum lipids at admission to predict LOS > 29 days

**Figure 3:**
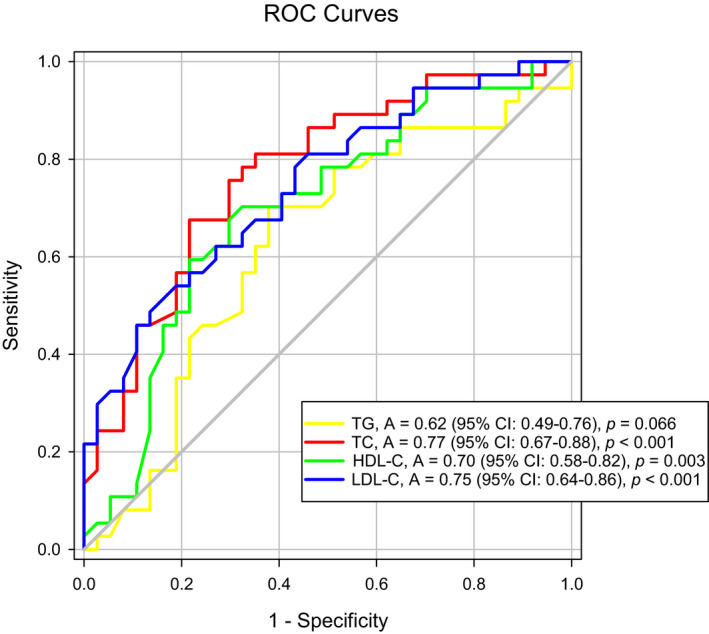
ROC curve analyses of discriminative values of serum lipids at admission for LOS > 29 days ROC curve, receiver operating characteristic curve; AUC, area under curve; CI, confidence interval

ROC curve analyses were chosen to evaluate the discriminative values of serum lipids for LOS > 29 days. As shown in Figure 3, TC, HDL‐C, and LDL‐C showed certain discriminative ability for LOS > 29 days [AUC: 0.77 (95% CI: 0.67–0.88, *p* < .001), 0.70 (95% CI: 0.58–0.82, *p = *.003), and 0.75 (95% CI: 0.64–0.86, *p* < .001), respectively]. TG did not show discriminative ability (AUC: 0.62, *p* = .066).

### 3.5 Prognostic value of lipids for LOS

Maximum Youden's indexes were calculated to determine the optimal cutoff values of TC, LDL‐C, HDL‐C, and age. The following cutoff values were selected: TC, 3.75 mmol/L (sensitivity 0.68, specificity 0.78); LDL‐C, 1.70 mmol/L (sensitivity 0.46, specificity 0.89); and HDL‐C, 0.95 mmol/L (sensitivity 0.59, specificity 0.78).

Univariate COX analysis was shown in Table S1 In the multivariate Cox proportional hazards models, we adjusted for serum lipids, age, the history of CHD, and previous statin treatment; and laboratory indicators, including albumin, FBG, and lymphocyte count. Different multivariate modes were made to avoid the influence of TC and LDL‐C collinearity on the regression analysis. As shown in Table 3, TC < 3.75 mmol/L (adjusted HR = 0.632, 95% CI: 0.482–0.830, *p* < .001) or LDL‐C < 1.7 mmol/L (adjusted HR = 0.557, 95% CI: 0.409–0.759, *p* = .001) at admission was an independent predictor of LOS prolongation.

**Table 3:** Independent predictors for length of hospital stay

Variables	Cutoff value	Multivariate analysis	
		Adjusted HR (95% CI)	*p*‐value
Mode 1			
Age	60 years	2.252 (1.714–2.959)	<.001
TC	3.75 mmol/L	0.632 (0.482–0.830)	.001
Lymphocyte	0.8 × 10^9^/L	–	.818
Albumin	35.0 g/L	–	.747
FBG	6.1 mmol/L	1.490 (1.093–2.031)	.012
CHD	Yes/no	–	.135
Previous Statin treatment	Yes/no	0.273 (0.168–0.442)	<.001
Mode 2			
Age	60 years	2.311 (1.760–3.035)	<.001
LDL‐C	1.70 mmol/L	0.557 (0.409–0.759)	.001
Lymphocyte	0.8 × 10^9^/L	–	.793
Albumin	35.0 g/L	–	.687
FBG	6.1 mmol/L	1.535 (1.126–2.092)	.007
CHD	Yes/no	–	.092
Previous Statin treatment	Yes/no	0.258 (0.165–0.433)	<.001

Note

Abbreviations as Table 1,2.

CI, confidence interval; HR, hazard ratio.

## 4 DISCUSSION

Lipids have a close relationship with lung biology and the pathophysiology of viral infection, but few studies have paid attention to alterations in lipid profiles in the context of viral pneumonia. There is no specific information on the relationship between lipids and NCP in recent reports (Cao et al., 2020; Huang et al., 2020; Ruan et al., 2020; Zhang et al., 2020).

Our study mainly focused on the characteristics of serum lipids in patients with NCP; the most compelling results are as follows: (a) The mean levels of LDL‐C at admission were significantly lower in severe patients. (b) The levels of TC and LDL‐C at admission were negatively correlated with LOS. (c) The levels of TC, LDL‐C, and HDL‐C in severe patients gradually and significantly increased during subsequent tests. (d) LDL‐C < 1.7 mmol/L at admission was an independent risk factor for LOS prolongation.

It seems that our observational results of gradually increasing levels of TC, LDL‐C, and HDL‐C in successive tests were inconsistent with a previous report on acute infectious diseases, namely, the common lipid alterations including decreased levels of TC, LDL‐C, and HDL‐C and increased concentrations of triglyceride‐rich lipoproteins (Filippas‐Ntekouan et al., 2017). However, when compared with the average lipid levels (TC 4.70 mmol/L, LDL‐C 2.88 mmol/L, HDL‐C 1.35 mmol/L, and TG 1.49 mmol/L) in a national survey of 163,641 adults conducted by Liu *et al*. (Zhang et al., 2018), we found that the lipid levels (TC 4.00 mmol/L, LDL‐C 2.11 mmol/L, HDL‐C 0.89 mmol/L, and TG 1.11 mmol/L) at admission in our study were much lower, which is consistent with the previously mentioned report (Filippas‐Ntekouan et al., 2017).

As expected, compared with the LOS ≤ 29 days group, the mean concentrations of TC, LDL‐C, HDL‐C, and TG at admission were lower in the LOS > 29 days group, and the TC level (3.56 ± 0.80 mmol/L) reached the definition of hypocholesterolemia. This pattern was remarkably consistent with the results of the 2nd and 3rd tests for TC and LDL‐C. Our results of lower lipid levels in the LOS > 29 days group suggested a positive correlation between lower serum lipids and a longer hospital stay, namely, more severe or complicated illness. The increased concentrations of TC and LDL‐C in the LOS > 29 days group remained lower than those in the LOS ≤ 29 days group, which also indicated that lower cholesterol levels were correlated with the prolongation of LOS, that is, the later recovery from NCP.

Hypocholesterolemia was associated with signs of further worsening of the disease (Oster et al., 1981). Hypocholesterolemia is often an index of pathophysiological frailty and impending danger (Vyroubal et al., 2008). The results from the MONDO international study also showed that lipid levels were inversely associated with infection‐related and all‐cause mortality (Kaysen et al., 2018). The mechanisms of lower lipid profiles in patients with NCP, particularly in severe critical patients, are not well known. The increased consumption of cholesterol associated with pulmonary surfactant synthesis to fight infection and viral replication may be one of the reasons. Inadequate nutritional intake and incapacity to synthesize to meet requirements in weak and severe patients with NCP may be the other main reasons.

We also found that in severe cases, lipid levels were gradually and significantly increased during the following 2nd (except for LDL‐C) and 3rd tests. To the best of our knowledge, this increasing trend was not reported in a previous study. Progressive increases in cholesterol may be considered a marker of reversal of critical illness and of patient recovery, but increasing cholesterol may induce or worsen underlying atherosclerotic cardiovascular diseases such as stroke or myocardial infarction. It is worth noting that the mean age of patients was 55.04 ± 15.87 years, and 25.4%, 12.5%, and 6.5% of patients had hypertension, diabetes, and CHD, respectively. A report from Wang et al. showed that 25% of patients with COVID‐19 in the intensive care unit had cardiovascular disease, and 58% had hypertension (Wang et al., 2020). The predisposition to acute cardiac complications related to underlying atherosclerotic cardiovascular disease may significantly increase the severity of COVID‐19 in susceptible individuals (Vuorio et al., 2020).

Based on our results, continually monitoring the alterations of lipids in elderly patients with concomitant diseases appears to be extremely important. First, low cholesterol levels at admission or decreasing cholesterol levels during hospitalization may indicate prolongation of hospital stay or even worse outcomes. In addition, uncontrolled gradual increases in cholesterol levels in NICP‐19 patients with underlying illnesses or risk factors may facilitate major adverse cardiovascular events. Therapeutic strategies should be carefully evaluated on an individual basis to modulate the concentration of cholesterols. At present, the concrete resolution for hypocholesterolemia is the rapid recovery of the underlying illness; the approach of supplying dietary cholesterol needs more investigation. Cholesterol‐lowering can be achieved by statin therapy. Statins not only reduce atherosclerotic complications but also reduce inflammatory reactions (Mach et al., 2020). Statin therapy may confer benefits in patients with pneumonia (Chopra & Flanders, 2009) and restrict the ability of the influenza virus to generate lipid droplets and severely suppress the replication of the virus (Episcopio et al., 2019). In our study, only 8.1% of patients used statin therapy; in the multivariate Cox hazards model, statin therapy seemed to show a protective effect reflected by a shortened LOS (Table 3). However, it was challenging to show a beneficial effect due to the limited sample size of patients using statins. Whether statins are used depends on cholesterol levels and relevant cardiovascular comorbidities; the balance of benefits and harm needs careful individual evaluation.

The observation of hypolipidemia at admission in the LOS > 29 days group guided us to explore whether lipids could have prognostic value in patients with NICP‐19. The most commonly reported predictors of severe prognosis in patients with NICP‐19 included age, sex, features derived from computed tomography scans, CRP, lactic dehydrogenase, lymphocyte count, and albumin (Wynants et al., 2020). In our study, covariates included not only serum lipids and the clinical features mentioned in the previous literature but also statin treatment. The adjusted HR still showed that TC or LDL‐C at admission was an independent predictor of LOS. In fact, serum cholesterol levels and the variables included in the multivariate COX regression model roughly reflect the baseline nutritional status of patients at admission. The differences need to be further studied and discussed in a large number of clinical cases.

Our study has several limitations. First, some of the patients were transferred from cabinet hospitals and some of them had received medical therapies such as antivirals and/or antibiotics, so the lipid levels at admission may not truly reflect the original state of the infection. Second, to investigate the subsequent alterations in serum lipids during hospitalization, patients who did not undergo tests three times were excluded, so the sample may not represent the whole population in our wards, and the sample size may not be large enough in this study. Third, other lipid components, such as apoA, apoB, VLDL, and Lp(a), were not detected in all the patients due to laboratory limitations, and the alterations of TC, LDL‐C, HDL‐C, and TGs may not reflect the exact alterations of lipid metabolism.

## 5 CONCLUSIONS

In patients with NCP, serum lipid levels were low at admission. Levels of TC and LDL‐C at admission were negatively correlated with LOS. Lower serum levels of TC (<3.75 mmol/L) or LDL‐C (<1.7 mmol/L) at admission may act as an independent predictor of LOS prolongation. In severe cases, the gradual increase in TC, LDL‐C, and HDL‐C during hospitalization might indicate the gradual recovery of the disease.

## CONFLICT OF INTEREST

The authors declare that they have no conflicts of interest.

## ETHICAL APPROVAL

The study protocol conforms to the ethical guidelines of the 1975 Declaration of Helsinki. The study protocol was approved by the ethics institutional review board of Fujian Medical University Union Hospital (No. 2020KY028).

## Supporting information

Table S1
