# Supplementary material for: Alteration of lipid profile and value of lipids in the prediction of the length of hospital stay in COVID‐19 pneumonia patients
Source: Food Sci Nutr. 2020 Oct 27;8(11):6144–52. doi: 10.1002/fsn3.1907 (PMC7684619; doi:10.1002/fsn3.1907)
Supplement: Supplementary file 1 — Table S1 [file FSN3-8-6144-s001.docx]

**Supplementary Table 1.** Univariate COX analysis for length of hospital stay

|  |  | **Univariate analysis** | |  |
| --- | --- | --- | --- | --- |
| **Variables** | **Cut-off value** | **HR (95% CI)** | ***p*-value** |  |
| Age | 60 years | 2.319 (1.778-3.024) | **<0.001** |  |
| Gender | male/female | 0.449 (0.504-1.355) | 0.826 |  |
| TC | 3.75 mmol/L | 0.581 (0.446-0.757) | **<0.001** |  |
| LDL-C | 1.70 mmol/L | 0.569 (0.421-0.771) | **<0.001** |  |
| HDL-C | 0.95 mmol/L | 0.801 (0.621-1.033) | 0.087 |  |
| Lymphocyte | 0.8×10^9^/L | 0.677 (0.481-0953) | **0.025** |  |
| Hemoglobin | 120 g/L | 0.858 (0.666-1.105) | 0.234 |  |
| AST | 40 U/L | 1.443 (0.992-2.098) | 0.055 |  |
| ALT | 40 U/L | 0.903 (0.661-1.232) | 0.519 |  |
| Albumin | 35.0 g/L | 0.543 (0.391-0.756) | **<0.001** |  |
| FBG | 6.1 mmol/L | 1.767 (1.302-2.398) | **<0.001** |  |
| Urea | 7.4 mmol/L | 1.440 (0.935-2.217) | 0.098 |  |
| Serum creatinine | 135 μmol/L | 1.790 (0.665-4.818) | 0.249 |  |
| Hypertension | yes/no | 1.027 (0.770-1.370) | 0.856 |  |
| Diabetes | yes/no | 1.187 (0.814-1.730) | 0.374 |  |
| CHD | yes/no | 0.598 (0.359-0.996) | **0.048** |  |
| Previous Statin treatment | yes/no | 0.433 (0.273-0.687) | **<0.001** |  |

Abbreviations as Table 1,2.

CI, confidence interval; HR, hazard ratio.
